# Effect of TET inhibitor on bovine parthenogenetic embryo development

**DOI:** 10.1371/journal.pone.0189542

**Published:** 2017-12-21

**Authors:** Jian Zhang, Sheng Zhang, Yutian Wang, Hui Cheng, Linlin Hao, Yanhui Zhai, Zhiren Zhang, Xinglan An, Xiaoling Ma, Xueming Zhang, Ziyi Li, Bo Tang

**Affiliations:** 1 College of Veterinary Medicine, Jilin University, Changchun, Jilin, China; 2 Academy of Translational Medicine, First Hospital, Jilin University, Changchun, Jilin, China; 3 Oncology Department, Second Hospital, Jilin University, Changchun, Jilin, China; Peking University Third Hospital, CHINA

## Abstract

DNA demethylation catalysed by the ten-eleven translocation (TET) protein is an important step during extensive global epigenetic reprogramming in mammals. However, whether TET proteins play a key role in DNA demethylation during the development of bovine pre-implanted embryos is still unclear. In this study, we utilized dimethyloxallyl glycine (DMOG), a small-molecule inhibitor of the TET protein, to impede the enzymatic activity of TET and explore subsequent effects on bovine parthenogenetic embryo development. We first detected the expression of the TET family, consisting of TET1, TET2 and TET3, in bovine MII stage oocytes and found that *TET3* is more highly expressed than *TET1* and *TET2*. Treatment with 1 mM DMOG increased 5mC levels (30.4% vs 79.8% at the 8-cell stage for satellite I, 25.3% vs 40.6% at the 8-cell stage for α-satellite, 20.5% vs 73.5% at the blastocyst stage for satellite I and 16.6% vs 30.0% at the blastocyst stage for α-satellite) at every bovine parthenogenetic embryo developmental stage. At the same time, DNA methylation level of satellite DNA and pluripotency gene promoters increased significantly. Real-time PCR analysis results indicated that the transcription levels of *NANOG* and *OCT-4* decreased in the DMOG-treated group. Furthermore, TET inhibition negatively affected blastocyst formation, resulting in a decline in the blastocyst rate (17.1 ± 1.3% vs 24.1 ± 0.6%); however, the percentage of apoptotic cells was significantly increased according to the results of a TUNEL assay. Additionally, expression levels of the apoptosis-related gene *BAX* were up-regulated, while the expression of *BCL-2* was down-regulated. In conclusion, these results support that TET plays important roles in bovine parthenogenetic embryo development by influencing DNA methylation reprogramming, gene expression and apoptosis.

## Introduction

DNA methylation is a crucial epigenetic modification that regulates genomic imprinting, gene expression, cellular differentiation and X-chromosome inactivation [1]. Moreover, genome-wide demethylation is essential for mammalian early embryogenesis. The basis for active and passive demethylation of the paternal and maternal genomes was unclear until the discovery of the ten-eleven translocation (TET) family, consisting of TET1, TET2 and TET3 [2]. TET proteins are Fe(II)- and 2-oxoglutarate-dependent enzymes that oxidize 5-methylcytosine (5mC) into 5-hydroxymethyl-cytosine (5hmC), 5-formylcytosine (5fC) and 5-carboxycytosine (5caC) [3].

In early mammalian embryos, epigenetic reprogramming dynamics have been detailed by immunofluorescence staining and single-cell DNA-methylation analysis [4], although most 5mC immunofluorescent signals are believed to correspond to multiple-copy repetitive regions [5]. DNA methylation is necessary for the process of genomic imprinting, and TET-mediated oxidation of 5-methylcytosine is also important for modulating signal pathways by promoting demethylation [6]. In mice, *TET/TET2* gene knockout did not affect the expression of pluripotency factors or ESC differentiation [7, 8], and mice developed normally in terms of reproductive capacity [9, 10]. In addition, *TET*3 knockout resulted in the death of mice after birth, and *TET3* was found to be involved in the development of multiple tissues during embryogenesis [11].

Small molecule inhibitors of 2-oxoglutarate (2-OG)- and Fe(II)-dependent dioxygenases include compounds that are structural analogues of the 2-oxoglutarate co-factor required for these enzymes to function [12]. One of these, DMOG, is a non-specific 2-OG-dependent dioxygenase inhibitor [13]. In mice, treatment of embryos with 1 mM DMOG from the germinal vesicle (GV) to blastocyst stage effectively blocks the activity of TET enzymes in vitro [14].

Parthenogenesis is defined as an embryo developing from an unfertilized oocyte though artificial activation. Parthenogenetic (PA) embryos lack paternally expressed genes, thus representing an important biological research model. PA embryos have been used for different types of research, including the investigation of fertilization and the imprinting process [15, 16], for co-transfer with cloned embryos as a key step in somatic cell nuclear transfer [17, 18], to test new embryo technologies [19] and to assess the quality of oocytes matured in *vitro* [15]. In addition, several epigenetic modifications (DNA methylation and histone modification) have been further explored and may play important roles during the development of parthenogenetic embryos [20].

Together, previous studies have shown that DNA methylation plays a critical role in early embryonic development. However, whether TET proteins participate in DNA methylation reprogramming and the mechanism by which TET proteins function in bovine parthenogenetic embryos are not well understood. Therefore, in this study, we utilized a small molecular inhibitor of TET, DMOG, and investigated its effects on bovine parthenogenetic embryo development by assessing DNA methylation reprogramming, gene expression and apoptosis.

## Materials and methods

Unless otherwise specified, chemicals and reagents were obtained from Sigma-Aldrich.

### Ethics statement

All experimental materials and procedures utilized in this study were reviewed and approved by the Animal Welfare and Research Ethics Committee at Jilin University

### *In vitro* maturation of bovine oocytes

Bovine ovaries were collected from a local abattoir and transported to the lab in 0.9% NaCl supplemented with 200 IU/ml penicillin at 36–37.5°C within 3–4 h. Cumulus-oocyte complexes (COCs) were extracted from the follicle using a 15-ml syringe attached to an 18-gauge needle. COCs) with at least three uniform layers of compact cumulus cells and a uniform cytoplasm were washed and cultured at 38.5°C in 100 μl of mature medium(TCM-199 supplemented with sodium bicarbonate, 10% foetal bovine serum, 1 μg/ml follicle-stimulating hormone and 10μg/ml luteinizing hormone) in a humidified 5% CO_2_ incubator for 22–24 h. Oocytes with the first polar body were considered to be mature.

### Parthenogenetic activation of bovine oocytes

Oocytes with the first polar body were treated with 5 mM ionomycin for 5 min and then disposed in 2 mM 6-dimethylaminopurine in synthetic oviduct fluid (Sofaa) containing 10% FBS for 4 h at 38.5°C with 5% CO_2_ in a 95% humidified atmosphere. After activation stimulation, the oocytes were then washed in Sofaa 2–3 times and randomly divided into two groups, the experimental group and the control group. The control group containing 25–30 mature oocytes was cultured in Sofaa-medium at 38.5°C in a humidified 5% CO_2_ incubator. For the experimental group, TET protein inhibition was performed in Sofaa medium with DMOG (Sigma-Aldrich) diluted in PBS buffer; the oocytes were incubated with drug in Sofaa containing 6-dimethylaminopurine for 4 h. The cleavage and blastocyst rates of the bovine parthenogenetic embryos were calculated at 2 d and 7 d after culturing, respectively.

### Immunofluorescence (IF) staining

Bovine oocytes/embryos were picked out and washed 2–3 times in PBS-PVA. The zona pellucida (ZP) was removed with 0.5% Pronase in PBS-PVA at 38.5°C for 5 min. The ZP-free oocytes/embryos were lightly washed in PBS-PVA and fixed in 4% paraformaldehyde for 30 min at 37°C. After fixation, they were washed in PBS-PVA 2–3 times and permeabilized in PBS with 0.2% Triton X-100 for 30 min. DNA was subsequently denatured using 4 N HCl for 20 min at 37°C and then neutralized with 100 mM Tris-HCl buffer for 15 min. Next, the ZP-free oocytes/embryos were incubated for 1 h at room temperature in PBS solution (3% BSA and 0.2% Tween-20 in PBS) to block non-specific binding sites. Primary antibodies targeting 5mC (diluted 1:200; Eurogentec) and 5hmC (1:200; Active Motif) were incubated with the oocytes/embryos overnight at 4°C, and the oocytes/embryos were washed three times in PBS-PVA. Alexa Fluor 488 (1:200; Invitrogen) and Alexa Fluor 594 (1:200; Invitrogen) were used to detect the primary antibodies for 2 h at 37°C in the dark. All samples were mounted between a cover slip and a glass slide supported by four columns of a mixture of petroleum jelly and paraffin (9:1) and immediately observed under a fluorescence microscope(Nikon, Tokyo, Japan). Images of the control and experimental groups were captured using the same microscope settings and exposure time. At least 6 embryos at each development stage were analysed. The evaluation of average fluorescence intensity in embryos was performed using image analysis software[21].

### RNA isolation, RT-PCR and qRT-PCR

RNA was extracted from at least eighty bovine oocytes or forty blastomeres of parthenogenetic embryos at different stages using the RNeasy Mini Kit (Qiagen, Hilden, Germany). Reverse transcription was performed using the 1st-Strand cDNA Synthesis kit (Promega, Madison, WI, USA) according to the manufacturer’s guidelines. Primers used for qRT-PCR analysis are presented in S1 Table. Quantitative amplification of cDNA was performed in 96-well optical reaction plates using SYBR Premix Ex TaqTM reagents (TaKaRa) and a LightCyclerR 96 Real-Time PCR System (Roche). The qRT-PCR mix(20 μl) contained 10 μl SYBR green premix, 2 μl of cDNA, 6.8 μl of RNase-free water, 0.6 μl each of the forward and reverse primers (0.5 μM) for each gene. The following programme was used to amplify all genes: 30 s of denaturation at 95°C, 40 cycles of PCR for quantitative analysis (95°C, 5 s and 60°C, 30 s), one cycle for melting curve analysis (95°C for 5 s, 60°C for 1 min, 95°C for 1 s) and cooling at 4°C. The relative expression levels of target genes were calculated using the 2^−ΔΔCT^ method[22]. The qRT-PCR analysis were performed three times for each sample. Values were normalized to bovine GAPDH mRNA.

### Sodium bisulfite genomic sequencing

Bisulfite sequencing was used to analyse locus-specific DNA methylation of oocyte and parthenogenetic blastocysts as described [23]. Briefly, at least fifty 8-cell or five parthenogenetic blastocysts were disposed with 20 μl of lysis solution (10 mM Tris-HCl, pH 7.6, 10 mM EDTA, 1% SDS, and 20 μg/μl of proteinase K in ddH_2_O) for 2 h at 37.5°C. The materials were subsequently boiled in a water-bath for 5 min, chilled in ice-water and quickly spun down, and then 4 μl of 2 M NaOH (working concentration 0.3 M NaOH) was added for 15 min at 50°C. The samples were blended with 2 volumes of 2% low melting point agarose and transported into chilled mineral oil to form beads. Then, freshly prepared bisulfite solution mixture (2.5 M sodium metabisulfite and 125 mM hydroquinone, pH 5) was used to transform the beads for 4–5 h in the dark and covered with mineral oil at 50°C. Reactions were discontinued by washing with 1 ml of Tris-EDTA buffer (pH 8.0) for 4 × 15 min. After desulfonation in 500 μl 0.2 M NaOH for 2 × 15 min, the beads were washed with 1 ml Tris-EDTA buffer for 3 × 10 min and water for 2 × 15 min, then used for PCR (*satellite I*, *α-satellite*, *H19*, *NANOG*). The primer sequences are listed in S1 Table. The purified PCR fragments were then cloned into a Pmd^™^19-T vector for sequencing (TaKaRa, Japan). The PCR amplifications and subsequent sequencing were performed three times for each sample. At least 10 clones per gene were sequenced. DNA methylation situations from 0% to 20% was considered as low DNA methylation level, from 21% to 50% was considered as moderate DNA methylation level, from 51% to 100% was considered as high DNA methylation level in our bisulfite sequence analysis.

### Statistical analysis

At least three technical replicates and biological replicates were performed for each data analysis. All statistical analyses were carried out with SPSS 18.0 for Windows software (SPSS Inc., Chicago) using one-way ANOVA. A value of *P <* 0.05 was considered statistically significant, and *P <* 0.01 was considered highly significant.

## Results

### The influence of DMOG on the development of bovine parthenogenetically activated embryos

The experimental groups were treated with 500 μM, 1000 μM, 1500 μM DMOG and the control group lacked DMOG treatment. When embryos were treated with 500 μM, the cleavage rate and blastocyst rate were unchanged compared to control group (*P <* 0.05). No influence on the cleavage rate (96.3 ± 1.2%) or 8-cell rate (57.1 ± 1.9%) was found from treatment with 1000 μM DMOG; however, the blastocyst rate (17.1 ± 1.3%) was insignificantly lower than that of the control group (24.1 ± 0.6%). Embryos treated with 1500 μM DMOG failed to develop into blastocysts (Table 1). Therefore, we selected 1000 μM as the final concentration for further research.

**Table 1:** Embryo cleavage rates and blastocyst rates following culture supplemented with DMOG.

Group	No. of embryos examined	No. of embryos cleaved (%)	No. of embryos developed to the 8-cell stage (%)	No. of embryos developed to the blastocyst stage (%)
Control (0 μM)	182	174 (95.9 ± 1.6)^a^	99 (56.9 ± 3.1)^a^	42 (24.1 ± 0.6)^a^
DMOG(500 μM)	191	183 (96.1 ± 0.9)^a^	106 (58.3 ± 2.3)^a^	47 (25.8 ± 0.7)^a^
DMOG(1000 μM)	188	181 (96.3 ± 1.2)^a^	102 (57.1 ± 1.9)^a^	31 (17.1 ± 1.3)^b^
DMOG(1500 μM)	184	176 (95.5 ± 1.3)^a^	41 (23.8 ± 2.5)^b^	none

Data are the mean ± SEM of at least three trials.

^a,b^ Different superscript letters within the same column express significant differences(*P <* 0.05).

### Expression of the *TET* gene family in bovine parthenogenetic preimplantation embryos

**Fig 1:**
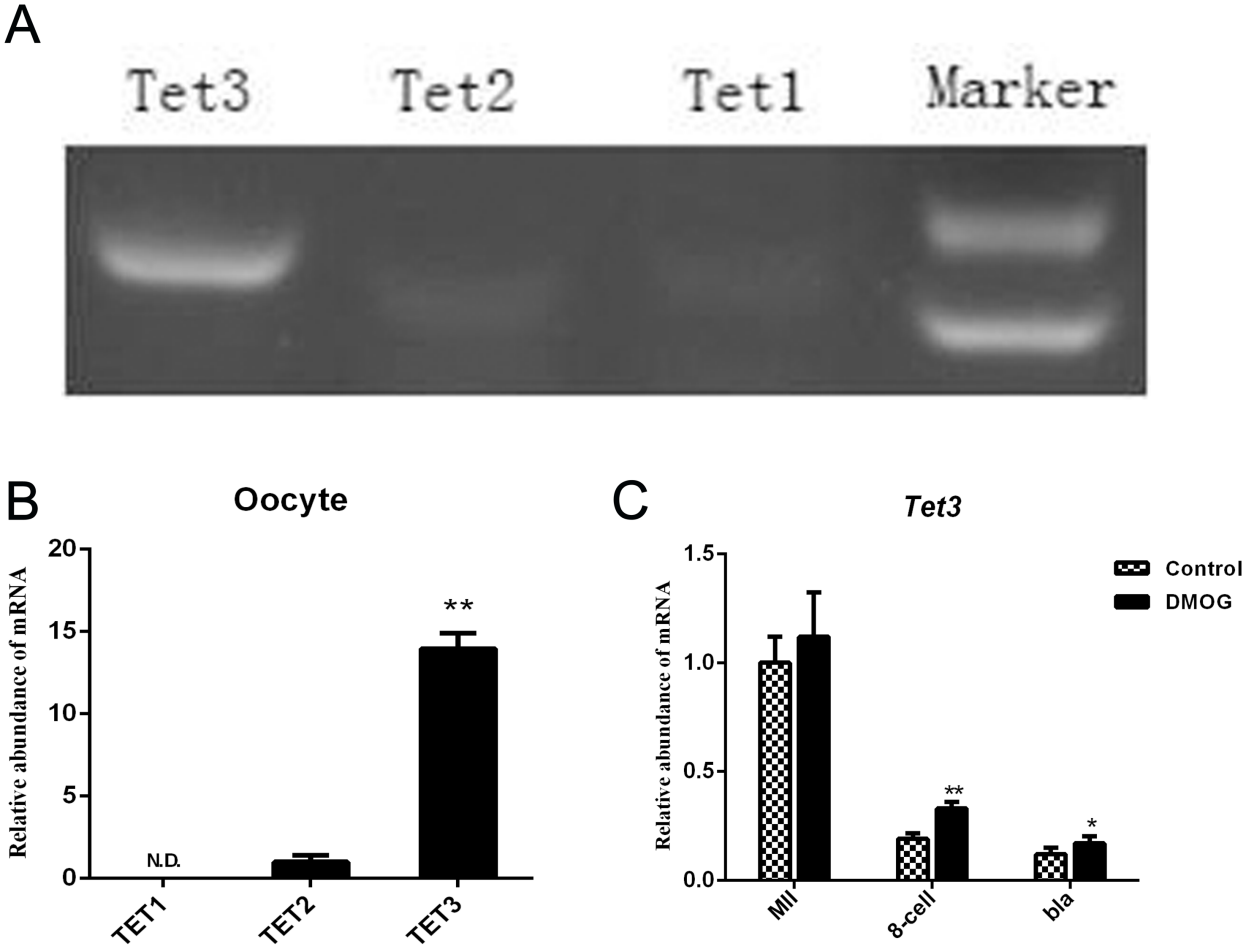
Relative abundance of TET protein transcripts in bovine preimplantation embryo stages. (A) Semiquantitative PCR indicated the expression of the *TET* gene family in bovine MII oocyte stage. (B) mRNA expression of the TET gene family in the bovine MII oocyte stage detected by qRT-PCR. TET2 gene expression levels were used for sample normalization (expression set to 1). (C) The relative abundance of *TET3* transcripts at the 8-cell and blastocyst stages in the control and DMOG groups. *TET3* expression levels in MII oocytes were used for sample normalization (expression set to 1). The results are shown as the mean ± standard deviation from three independent experiments; *P < 0.05, ** *P <* 0.01 compared with the control group.

The results revealed that transcripts of *TET1*, *TET2* and *TET3* were differentially expressed in bovine MII stage oocytes. *TET3* showed significantly high expression in the bovine MII stage oocytes compared to *TET1* and *TET2* (*P* < 0.01) (Fig 1A and 1B). The qRT-PCR results showed that treatment with DMOG (1 mM) had no influence on the expression of *TET3* (*P* > 0.05), but it significantly increased *TET3* expression in 8-cell stage (*P* < 0.01) and blastocyst stage embryos (*P* < 0.05) (Fig 1C).

### IF staining for 5-mC and 5-hmC in control and DMOG group embryos

**Fig 2:**
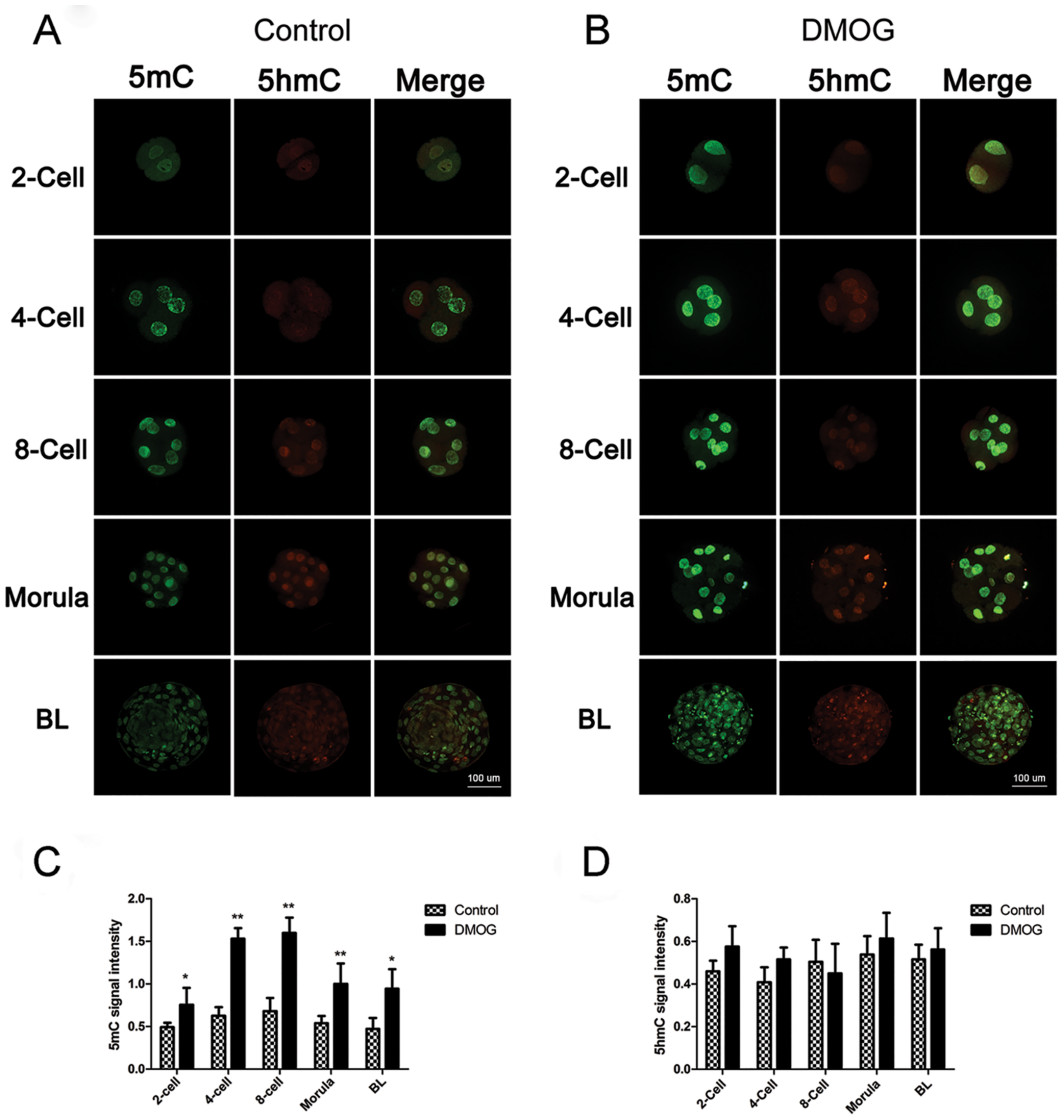
IF staining of 5-mC and 5-hmC at different stages in bovine parthenogenetic embryos. (A, B) 5mC and 5hmC staining in control and DMOG group embryos, respectively. (C, D) 5-mC and 5-hmC signal intensities were measured at each developmental stage. The results expressed as the mean ± standard deviation of five independent experiments; *P < 0.05, * * *P <* 0.01, compared with blastocysts in the control group. BL: blastocyst.

IF staining was used to examine global DNA methylation level in 2-cell, 4-cell, 8-cell, morula and blastocyst stage parthenogenetic embryos (Fig 2A and 2B). IF staining results showed that treatment with DMOG significantly increased 5-mC levels in bovine parthenogenetic embryos compared with control group (Fig 2C). However, no significant difference in 5-hmC signal levels was observed between the control and DMOG groups (Fig 2D).

### Methylation of satellite I, α-satellite

**Fig 3:**
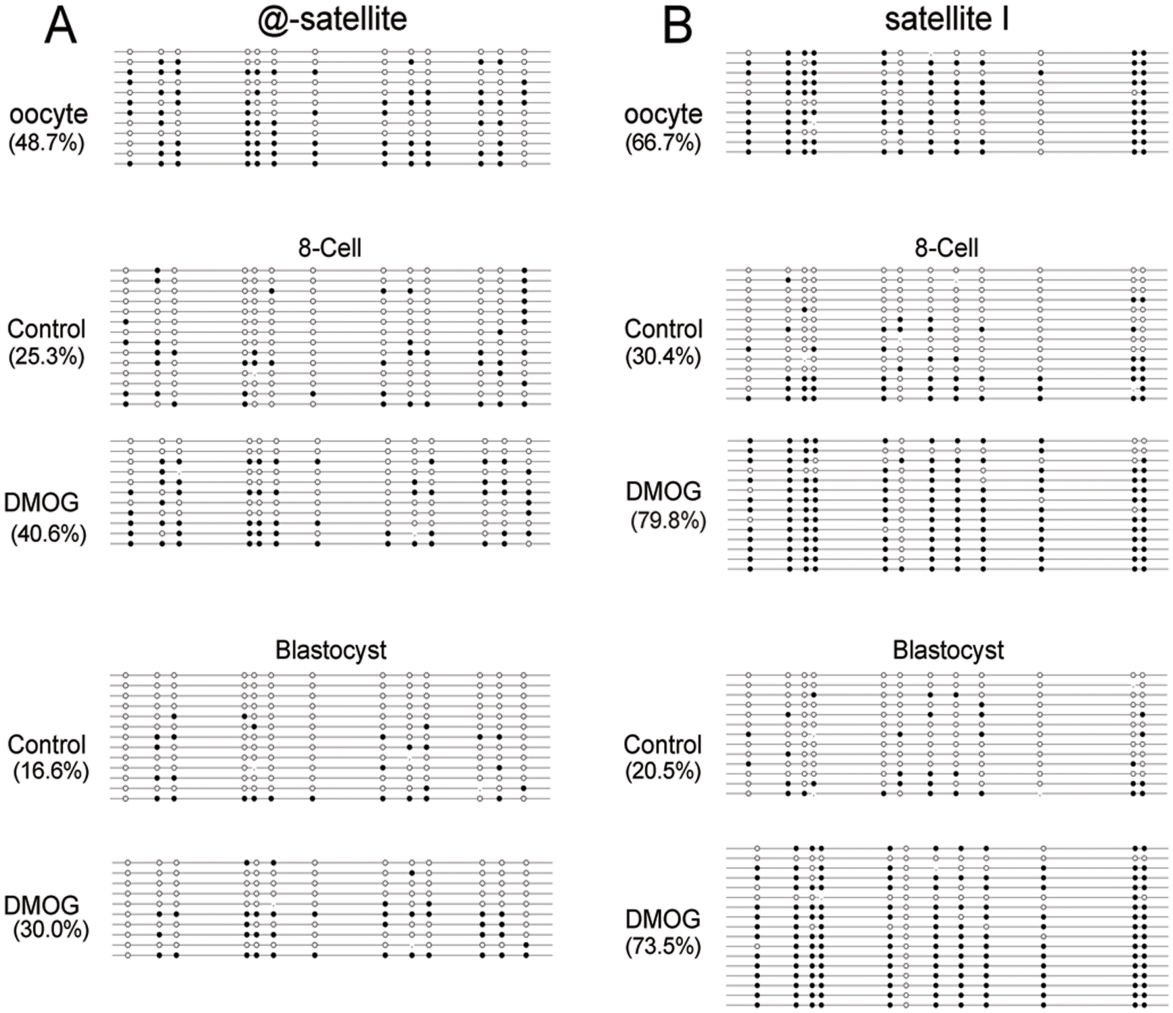
Dynamic DNA methylation profiles of satellite I and α-satellite. (A) Bisulfite sequencing analysis of α-satellite in oocytes, 8-cell and blastocysts of control and DMOG-treated embryos. (B) Bisulfite sequencing analysis of satellite I in oocytes, 8-cell and blastocysts of control and DMOG-treated embryos. The filled (black) circles indicate methylated cytosines, the unfilled (white) circles correspond to unmethylated cytosines. The numbers express the proportion of methylated cytosines occupied at all of the CpG sites.

To further verify the relevance of TET-mediated 5mC oxidation in bovine parthenogenetic embryos, we chose repeat elements to test DNA methylation status. Specifically, we detected the DNA methylation status of satellite I and α-satellite promoter regions using sodium bisulfite genomic sequencing in oocytes, and the sequencing results showed that α-satellite and satellite I had high DNA methylation level in MII oocytes (Fig 3A and 3B). Subsequently, we analysed DNA methylation level of these repeat genes in 8-cell embryos and blastocysts. In the control group, parthenogenetic embryos displayed a moderate DNA methylation level but significantly higher (*P <* 0.05) in comparison with the DMOG group at the corresponding stage, showing 30.4% vs 79.8% at 8-cell for satellite I, 25.3% vs 40.6% at 8-cell for α-satellite, 20.5% vs 73.5% at blastocyst for satellite I and 16.6% vs 30.0% at blastocyst for α-satellite, respectively.

### Methylation of H19

**Fig 4:**
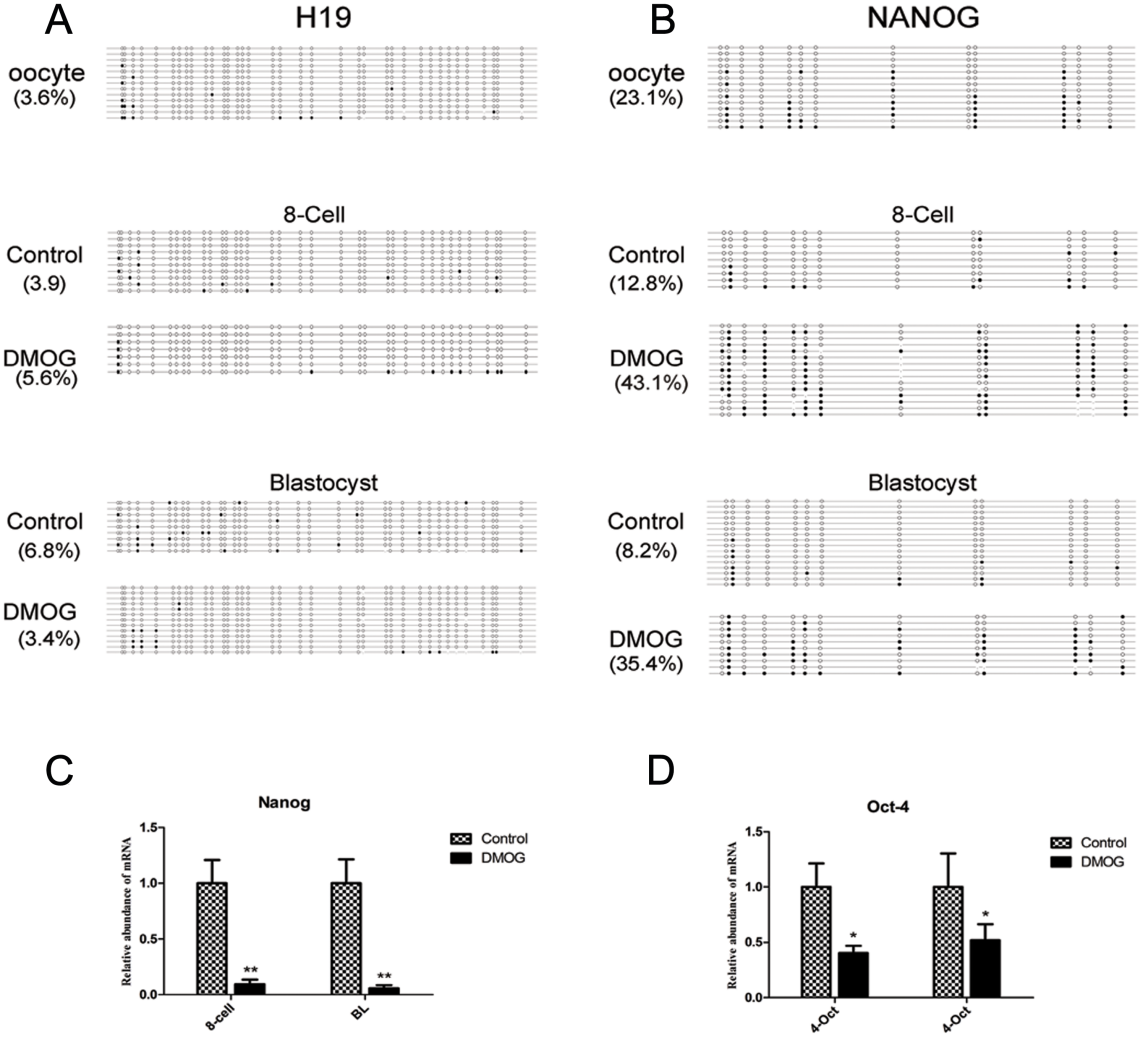
Dynamic DNA methylation profiles of several genes and the expression of pluripotency genes. (A) Methylation level of the *H19* gene at the 8-cell and blastocyst stages in control and DMOG group. (B)Methylation level of the *NANOG* gene at the 8-cell and blastocyst embryo stages of the control and DMOG group. The filled (black) circles indicate methylated cytosines, the unfilled (white) circles correspond to unmethylated cytosines. The numbers express the proportion of methylated cytosines occupied at all of the CpG sites. (C,D) mRNA abundance of the genes *NANOG* and *OCT-4* in the 8-cell and blastocyst stages of the control and DMOG group detected by qRT-PCR. The expression level of parthenogenetic embryos in the control group was used for sample calibration (expression set to 1). The results shown are the mean ± standard deviation from three independent experiments; **P < 0.01, compared with blastocysts in the control group.

Imprinted genes have been shown to play a pivotal role in embryonic growth and development. Thus, we chose one DMR region of *IGF2/H19* as a representative imprinted gene locus to detect DNA methylation states in control and DMOG group embryos. The results revealed that the DMR of *IGF2/H19* was hypomethylated in MII oocytes (3.6%). The lower level of DNA methylation was maintained in 8-cell embryos (3.9%) and blastocysts (6.8%), and no difference of DNA methylation was observed in the DMOG group, showing 5.6% at 8-cell and 3.4% at blastocyst, respectively (Fig 4A).

### Methylation and expression of pluripotency genes

We next asked whether TET suppression was correlated with gene expression in bovine parthenogenetic embryos. To this end, we analysed the DNA methylation status and mRNA expression levels of pluripotency gene promoter regions in oocytes, 8-cell stage and blastocyst stage parthenogenetic embryos. The BSP results show that DNA methylation levels of the *NANOG* promoter region were from 12.8% to 43.1% in 8-cell stage and from 8.2% to 35.4% in blastocysts (Fig 4B). Furthermore, the transcription levels of pluripotency genes *NANOG* and *OCT-4* were significantly higher in the control group than in the DMOG group in 8-cell and blastocyst embryos (*P <* 0.01) (Fig 4C and 4D). Together, our results revealed that TET inhibition increased DNA methylation levels of pluripotency gene promoter regions and decreased mRNA expression levers of pluripotency genes.

### Effects of DMOG treatment on cell apoptosis in bovine parthenogenetic preimplantation embryos

**Fig 5:**
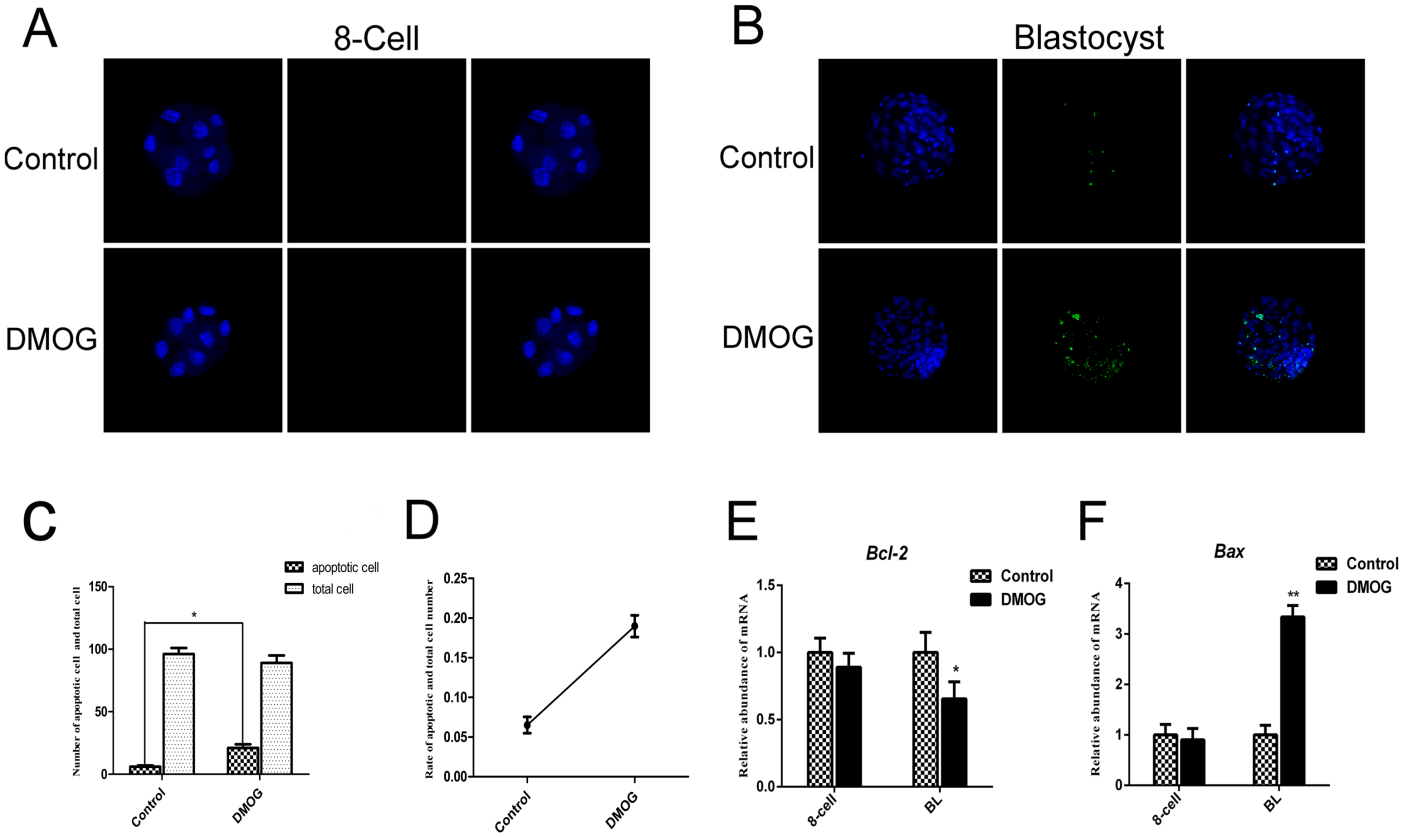
Assessment of apoptosis in control and DMOG-treated embryos. (A, B) TUNEL assay with 8-cell and blastocyst stage embryos from the control and DMOG groups. Fragmented nuclei (green) are apoptotic. Each sample was counterstained with Hoechst 33258 to visualize DNA (blue). (C) Number of apoptotic cell and total cells in the blastocyst stage for the control and DMOG groups. (D) Index between apoptotic cell number and total cell number. (E, F) qRT-PCR for *BCL-2* and *BAX* at the 8-cell and blastocyst stages for the control and DMOG groups. The expression levels of 8-cell embryos in the control group were used for sample normalization (expression set to 1). The results are shown as the mean ± standard deviation from three independent experiments; *P < 0.05, **P < 0.01 compared with blastocysts in the control group.

Then, we examined apoptotic cell numbers using a TUNEL assay performed on 8-cell and blastocyst stage embryos. IF staining results showed that there was almost no difference in apoptotic cell numbers in 8-cell stage embryos between the control and DMOG groups (Fig 5A). Additionally, total cell numbers did not differ in blastocyst stage embryos between the control and DMOG groups (Fig 5C). However, the average total number of apoptotic cells was significantly higher in the DMOG group than in control blastocysts (7.1 vs 18.6; Fig 5B and 5D). The mRNA levels of the representative apoptotic genes *BCL-2* and *BAX* were confirmed by qRT-PCR and found to be unchanged in 8-cell stage embryos. However, the group treated with DMOG exhibited a lower expression of *BCL-2* (*P* < 0.05) (Fig 5E) and significantly higher mRNA levels of *BAX* (*P* < 0.01) (Fig 5F) than the control groups.

## Discussion

During early embryonic development, the mammalian genome is subject to extensive epigenetic reprogramming. In previous studies, TET3 was primarily expressed in oocytes and zygote stage, indicating that TET3 was directly involved in active DNA demethylation processes in mouse early embryonic development [11]. Porcine research showed that TET1 and TET2 are highly expressed and TET3 is low in IVF embryos and SCNT embryos, suggesting that TET1 is involved in active DNA demethylation in porcine early embryonic development [24]. However, which TET family members play leading roles in DNA demethylation during early embryonic development and the relevance of the TET protein in bovine early embryonic development have remained unknown.

In this study, we utilized DMOG to inhibit the enzymatic activity of TET and investigate the effect of TET on bovine parthenogenetic embryo development [25]. Our research showed that compared to control embryos, TET with 1 mM DMOG led to similar cleavage and 8-cell rates but a significantly lower blastocyst rate. We therefore chose this concentration (1 mM) for use with bovine early embryos. QRT-PCR revealed high expression of *TET3* in the bovine MII oocyte stage compared to *TET1* and *TET2*, which is similar to results in mouse embryos [11] and is not like the study on porcine embryos [24]. In addition, we found that treatment with a TET inhibitor (DMOG) increased the transcription levels of *TET3* in 8-cell and blastocyst stage parthenogenetic embryos, which may be caused by the effects of intracellular compensation and feedback effect.

Immunofluorescence analysis provides an indirect quantification of DNA methylation levels during the development of mammalian preimplanted embryos [26]. Here, strong staining by the 5mC antibody was detected in bovine parthenogenetic embryos from the 2-cell stage to the blastocyst stage. Treatment with DMOG led to a significant increase in the 5mC signal in different embryo stages, which indicates that maternal DNA in bovine parthenogenetic embryos is subject to TET-mediated oxidation of 5mC. Notably, treatment with DMOG does not significantly influence the IF intensity of maternal 5hmC signals, which may be the reason that 5hmC showed highly dynamic global changes in early cleavage stages [27], and 5hmC accumulation is also regulated by the activity of *Dnmt1* and *Dnmt3a* [14].

In mammals, repeat elements overlap the majority of the genome, and functional elements cover approximately 1.5% of the global genome [28]. Thus, the changes and dynamics of 5mC signal by IF staining primarily correspond to multiple-copy repetitive regions [5]. To provide evidence additional to our IF staining results, we utilized bisulfite sequencing technology to detect locus-specific DNA methylation states of α-satellite and satellite I during bovine preimplantation embryonic development. Our results showed that DMOG treatment obviously increased DNA methylation level of satellite I and α-satellite in 8-cell stage and blastocyst stage embryos. Considering these facts we further verified our results that maternal DNA in bovine parthenogenetic embryos undergoes TET-mediated oxidation of 5mC.

Genomic imprinting is a characteristic epigenetic phenomenon in mammals. The disruption of imprinted genes leads to abnormalities such as foetal overgrowth, and the DMRs at imprinted loci are faithfully maintained during the early preimplantation embryonic development of mice [29]. We found that the methylation status of *H19* DMR remained hypomethylation from the oocyte to blastocyst stage, and DMOG treatment did not alter this pattern. These results confirmed that the gametic and post-fertilization modifications in imprinted regions were separable events; similar results were also obtained in mice [30], and TET protein has no role in imprinted regions of maternal *H19* DMR in bovine parthenogenetic embryos.

It is known that pluripotency-related genes play essential roles in early embryonic development [31], and DNA methylation, a major epigenetic modification, is generally believed to regulate the expression of these pluripotency-related genes [32]. Considering that the DNA methylation level at promoters is related to gene expression [33], we first analysed DNA methylation levels at the promoter of pluripotency gene *NANOG*. DMOG treatment significantly increased the DNA methylation levels in the *NANOG* promoter region in 8-cell and blastocyst stage embryos. Additionally, treatment with DMOG decreased the mRNA transcription levels of *NANOG* and *OCT4* during early embryonic development. These results indicate that the maintenance of *NANOG* gene methylation levels is connected with TET activity, and the high methylation levels of the promoter region down-regulated the expression of the pluripotency gene *NANOG*.

Based on the results in the present study, we speculated that abnormal DNA methylation occurs for genes that cause cell apoptosis. In fact, the TUNEL assay results showed that DMOG treatment increased cell apoptosis during the blastocyst stage. Additionally, expression of the pro-apoptotic gene *BAX* was strongly up-regulated, while *BCL-2* expression was down-regulated following treatment with DMOG. In addition, DMOG had no effect on the expression levels of the pro-apoptotic gene *BAX* or the number of apoptotic cells at the 8-cell stage.

In conclusion, our results suggested that TET3 may be responsible for maternal active DNA demethylation, and TET-dependent DNA demethylation plays key roles in development, DNA methylation reprogramming, pluripotency-related gene expression and the apoptosis of bovine parthenogenetic embryos.

## Supporting information

S1 Table Primer sequences used for qRT-PCR and bisulfite sequencing PCR. (DOC)
