# Supplementary material for: Effect of TET inhibitor on bovine parthenogenetic embryo development
Source: PLoS One. 2017 Dec 21;12(12):e0189542. doi: 10.1371/journal.pone.0189542 (PMC5739418; doi:10.1371/journal.pone.0189542)
Supplement: S1 Table — (DOC) [file pone.0189542.s001.doc]

**Table S1**

Primers for qRT-PCR and Bisulfite-sequencing PCR.

| Primer Primer pair sequences (50 to 30)a Product size (bp) |
| --- |
| **qRT-PCR**  18S rRNA F:GACTCATTGGCCCTGTAATTGGAATGAGTC 87  R: GCTGCTGGCACCAGACTTG  Nanog F: AACAACTGGCCGAGGAATAG 193  R: AGGAGTGGTTGCTCCAAGAC  Oct4 F:GGCGCCAGAGGAAAGGATAC 173  R:AGAAGGGCAAACGATCAAGCA  Bax F:GTGCCCGAGTTGATCAGGAC 126  R: CCATGTGGGTGTCCCAAAGT  Bcl-2 F: AGCATCACGGAGGAGGTAGAC 141  R: CTGGATGAGGGGGTGTCTTC  **Bisulfite-sequencing PCR**  Nanog F: TTTTTTAATTATAATTTGATGGGGT 288  R: CTAACACACCTTAAATAAACAAACC  α-satellite F: AATAATTCCACATTCCRTAAAACCC 189  R: GATGTTTYGGGGAGAGAGG  Satellite I F: AATACCTCTAATTTCAAACT 211  R:TTTGTGAATGTAGTTAATA  H19 F: TTAAGGTTTTGGTTTTTGTTT 390  R: AACTTCAAAATTACCTCCTACC |

a F, forward; R, reverse.
